# Marked Efficacy of a Therapeutic Strategy in a Patient With Necrotizing Myopathy Associated With Anti-signal Recognition Particle (SRP) Autoantibodies

**DOI:** 10.7759/cureus.35471

**Published:** 2023-02-25

**Authors:** Murouj Almaghrabi, Shahad B Almatrafi, Asma Alzahrani, Mohammed Alharbi

**Affiliations:** 1 Department of Medicine and Surgery, College of Medicine, Umm Al-Qura University, Al-Abdia Main Campus, Makkah, SAU; 2 College of Medicine, Umm Al-Qura University, Al-Abdia Main Campus, Makkah, SAU; 3 Department of Neuroscience, King Abdullah Medical City, Makkah, SAU

**Keywords:** autoantibodies, therapeutic plasma exchange (tpe), plasma exchange, rituximab, therapeutic, signal recognition particle, necrotizing myopathy

## Abstract

Necrotizing autoimmune myopathy (NAM) is a rare muscular disorder characterized by severe proximal muscle weakness. Risk factors include statin use, malignancy, and connective tissue diseases. The current study presents the first case of NAM in Saudi Arabia in a 26-year-old female who presented with proximal upper and lower limb weakness, dysphagia, dysarthria, and dyspnea with no previous medical or surgical history and was not on medication. Targeted myopathic antibody analysis revealed antibodies to signal recognition particles (SRP), and the serum creatinine kinase level reached 9308 U/L. A diagnosis of NAM was made, and the patient was started on the management plan. We discussed an interesting case progression and adverse effect challenges, as well as the management of these difficult-to-treat conditions.

## Introduction

Necrotizing autoimmune myopathy (NAM), also known as immune-mediated necrotizing myopathy (IMNM), is a rare muscular condition with an unknown etiology in more than 50% of cases. A few risk factors have been identified, including statin use, malignancy, and connective tissue diseases (34%, 9.5%, and 4.2%, respectively) [1]. NAM is a subtype of idiopathic inflammatory myopathy characterized by symmetrical, proximal, subacute muscle weakness and a high creatine kinase level (CK) [2], as well as the muscle biopsy findings of pauci-immune necrosis and the absence of extra muscular manifestations [3,4]. NAM is known to be strongly associated with autoantibodies against signal recognition particles (SRP) [5] and 3-hydroxy-3-methylglutaryl-coenzyme A reductase (HMGCR) [6]. While other autoimmune myopathies such as dermatomyositis are strongly associated with other known autoantibodies including Mi-2, melanoma differentiation-associated gene 5 (MDA5), transcriptional intermediary factor 1 γ (TIF1 γ), and nuclear matrix protein 2 (NXP-2) [7]. In NAM, SRP antibody levels correlate with CK levels to determine the severity and prognosis of the patient's illness [5].

Inflammatory myopathies, including NAM, are usually managed with various treatment strategies, including steroids, intravenous immunoglobulin (IVIG), and other immunosuppressants, including methotrexate. Nevertheless, numerous reports have reported treatment resistance among NAM patients, and almost all patients require two or more immunosuppressants [8]. Given the correlation between pathogenic antibodies and disease severity in NAM, therapeutic plasma exchange (TPE) could aid in therapy by eliminating these antibodies. Additionally, the effectiveness of rituximab in treating SRP and HMGCR-associated NAM lends credence to the idea that the illness is primarily antibody-mediated [9,10]. In the current study, we present the case of a 26-year-old female patient who was diagnosed with NAM and showed marked improvement after an effective therapeutic strategy. This is the first case report of this kind in Saudi Arabia.

## Case presentation

A 26-year-old woman with no known medical or surgical history presented to the emergency department of our hospital last year complaining of a four-week history of dysphagia, dysarthria, dyspnea, and progressive, symmetrical, and proximal upper and lower limb weakness with a diurnal variation. She is not on any medication. Neurological examination of motor strength was graded as follows: bilateral proximal upper limb (UL): 3/5, bilateral distal UL: 5/5, bilateral proximal lower limb (LL): 3-/5, and bilateral distal LL: 5/5. The patient was wheelchair-bound. Other neurological examinations revealed normal findings. Laboratory investigation showed slightly elevated C-reactive protein (2.04 mg/dL), creatine kinase (CK) (peak 9308 U/L), as well as positive anti-SRP and anti-Mi2 alpha antibodies (Table 1). Electromyography (EMG) revealed positive spontaneous activities with myopathic motor unit potential (MUP). The patient underwent a computerized tomography (CT) scan of the kidneys, ureters, and bladder (KUB) as well as a mammogram, both of which revealed no evidence of malignancy.

**Table 1:** Laboratory parameters during the diagnostic work-up of the patient

Parameter	Result	Reference range (units)
Aspartate transaminase (AST)	224	8.0 – 43.0 (U/L)
Alanine transaminase (ALT)	145	12 – 78 (U/L)
C-reactive protein (CRP)	2.04	<0.5 (mg/dL)
Creatinine kinase (CK)	9308	29 – 168 (U/L)
Anti-nuclear antibodies (ANA)	1.01	Negative: <1
Anti-signal recognition particles (anti-SRP)	Positive	Negative
Anti-Mi2 alpha	Positive	Negative
Anti-Mi2 beta	Negative	Negative
Acetylcholine receptor antibody (ARA)	Negative	Negative
Ro-52	Negative	Negative

Treatment was started first with five doses of intravenous immunoglobulins (IVIg) (2 gm/kg/dose), and later on, the patient was administered rituximab. After 10 days of receiving rituximab, the patient came to the ER complaining of fever (39°C) associated with shortness of breath (SOB), nausea, diarrhea, bilateral shoulder pain, left wrist pain, and excessive worsening of upper and lower muscle weakness. Examination revealed the proximal power of bilateral UL at 2/5 and the proximal power of bilateral LL at 3/5. In addition, the patient had hypotonia and hyporeflexia. Thus, rituximab was withdrawn as the patient developed acute serum sickness as a side effect of the drug. The patient was started on high-dose steroids (a high dose for the patient herself; 1 mg/kg) with monthly therapeutic plasma exchange (TPE) and received a total of five cycles as well as mycophenolate mofetil 250 mg. Steroid doses were decreased gradually until they were stopped.

After completion of all TPE sessions, the serum CK level improved from 9308 U/L to 1515 U/L, and the anti-SRP antibody level decreased as well. Notably, dysphagia and dysarthria resolved. And the weakness improved remarkably as the patient was able to walk with a cane. The patient was discharged on mycophenolate mofetil 500 mg to be taken twice a day. At the latest follow-up in the outpatient clinic, the weakness improved dramatically, and the patient is now able to walk independently and climb stairs as well. Her CK levels have fallen to 98 U/L.

## Discussion

The current study reported the first case of NAM in Saudi Arabia, which was diagnosed and treated in King Abdullah Medical City, Makkah. The latest evidence by Kruse et al. in 2022 presented a review of the literature for similar reports; hence, they revealed a total of 25 patients involving seven countries in the United States, Australia, Japan, France, Ireland, Switzerland, and China [11]. All the included patients had a myopathy-related autoantibody, either SRP or HMG-CoA reductase (HMGCR), and were treated with multiple immunosuppressants before the administration of TPE. Following TPE, all patients exhibited reductions in serum CK level and at least transient, subjective improvements in strength and capability to perform daily activities. The activity of serum CK in NAM cases was also noted, where CK levels rise exponentially with disease activity and rise prior to the onset of weakness; yet, during treatment, CK levels fall prior to strength recovery.

SRP antibodies were first described in the 1980s [12] and are found in 39% of patients with NAM [13]. In fact, these autoantibodies are associated with a so-called "necrotizing myopathy," generally rapidly progressive and responsible for major muscle weakness [14,15], and were also recognized as an autonomous entity [16]. Other patients with NAM have HMGCR antibodies (26%), and the remaining 35% have other pathologic antibodies (e.g., ribonucleoprotein (RNP) or Ro) [13]. Patients without HMGCR or SRP antibodies may respond to treatment less favorably, have a more severe illness, or manifest extra-muscularly more frequently [17]. Our patient, who had SRP autoantibodies, had rapidly progressive severe symptoms of dysphagia, dysarthria, dyspnea, and progressive, symmetrical, and proximal upper and lower limb weakness. Even with a high dose of a slow-tapering steroid, our patient showed no remarkable improvement. This is consistent with a previous study by Miller et al., who described the incomplete response to corticosteroids among seven patients with anti-SRP antibodies [14]. Thus, it is known that a lack of response to corticosteroid therapy alone is common, and the addition of a single or combined immunosuppressant treatment is generally necessary [9]. Moreover, the value of the therapeutic strategy of combining corticosteroids, plasma exchanges, and rituximab is highlighted here. In the context of the progression of NAM disease, the ideal protocol and timing for TPE therapy introduction are still unknown. In the previous series [11], TPE treatment ranged from intensive short-term courses (one to five procedures) to prolonged outpatient tapers. Generally, greater muscle damage in NAM is associated with worse outcomes after treatment [18]. Cases of insufficient response to rituximab are also possible [19]. Although one of the particularities of this case report was the additional challenges upon receiving rituximab, our patient developed acute serum sickness, which required additional care and intervention; thus, physicians have to be sensitive regarding the possible adverse effects of medication when treating their patients.

## Conclusions

Necrotizing myopathy associated with anti-SRP autoantibodies treated with the combination therapy of steroids, therapeutic plasma exchange, and rituximab revealed remarkable effectiveness. The present case revealed an effective management strategy for those difficult-to-treat conditions. Although adverse effects are possible, physicians have to be aware of them. Furthermore, the study revealed a unique presentation of a 26-year-old female with unknown risk factors. Additional investigations are needed for a better understanding of the condition.
